# Determination of Formation Energies and Phase Diagrams of Transition Metal Oxides with DFT+*U*

**DOI:** 10.3390/ma13194303

**Published:** 2020-09-26

**Authors:** Daniel Mutter, Daniel F. Urban, Christian Elsässer

**Affiliations:** 1 Fraunhofer Institute for Mechanics of Materials IWM, Wöhlerstraße 11, 79108 Freiburg, Germany; daniel.urban@iwm.fraunhofer.de (D.F.U.); christian.elsaesser@iwm.fraunhofer.de (C.E.); 2 Freiburg Materials Research Center (FMF), University of Freiburg, Stefan-Meier- Straße 21, 79104 Freiburg, Germany

**Keywords:** transition metal oxides, density functional theory, DFT+*U*, materials modeling, phase diagrams

## Abstract

Knowledge about the formation energies of compounds is essential to derive phase diagrams of multicomponent phases with respect to elemental reservoirs. The determination of formation energies using common (semi-)local exchange-correlation approximations of the density functional theory (DFT) exhibits well-known systematic errors if applied to oxide compounds containing transition metal elements. In this work, we generalize, reevaluate, and discuss a set of approaches proposed and widely applied in the literature to correct for errors arising from the over-binding of the O_2_ molecule and from correlation effects of electrons in localized transition-metal orbitals. The DFT+*U* method is exemplarily applied to iron oxide compounds, and a procedure is presented to obtain the *U* values, which lead to formation energies and electronic band gaps comparable to the experimental values. Using such corrected formation energies, we derive the phase diagrams for LaFeO_3_, Li_5_FeO_4_, and NaFeO_2_, which are promising materials for energy conversion and storage devices. A scheme is presented to transform the variables of the phase diagrams from the chemical potentials of elemental phases to those of precursor compounds of a solid-state reaction, which represents the experimental synthesis process more appropriately. The discussed workflow of the methods can directly be applied to other transition metal oxides.

## 1. Introduction

Due to their exceptional electronic, magnetic, or optical properties [1], transition metal oxide (TMO) compounds are key components of many modern technologies. Next to many other applications, TMOs are utilized as active anode and cathode materials in Li- and Na-ion batteries [2,3,4,5] and as electrodes in solid-oxide fuel and electrolyzer cells [6,7,8] (SOFC and SOEC), where they enable the catalytic reactions with oxygen. The decisive functional parameters of a TMO, such as the catalytic activity and the electronic conductivity in the case of a solid-oxide cell electrode, can be tuned by varying the stoichiometry, e.g., by external doping or the incorporation of intrinsic lattice defects [9,10,11]. A phase diagram with respect to elemental reservoirs determines the ranges of the experimental synthesis conditions within which the desired compound can be stabilized and within which its composition can be varied without unwanted competing phases being formed.

In order to derive such a phase diagram theoretically, formation energies need to be known of all compound phases, which consist of a subset of the involved elements. Highly accurate formation energies can, in principle, be calculated using the methods of density functional theory (DFT) [12]. However, the determination of formation energies of TMOs using common (semi-)local exchange-correlation approximations of DFT (namely local-density or generalized-gradient approximations, LDA or GGA) raises some fundamental issues leading to results that systematically deviate from the experimental values [13,14,15,16]:

- The molecular state is the natural reference phase for oxygen. The well-known over-binding of the O_2_ molecule in LDA and GGA [17] introduces an error in the formation energies of oxides. A correction scheme was proposed by Wang et al. [13], which builds on a comparison between the theoretical and experimental values of formation energies for a series of non-TM oxide compounds. In the Materials Project (MP) database [18], for example, this scheme is applied and denoted by the term *anion corrections*.
- The uncompensated electronic self-interaction imposed by approximate exchange-correlation functionals immanent in DFT methods, especially in the case of TMOs, where strongly correlated TM-*d*-electrons form the valence band, leads to incorrect total energies and underestimated band gaps. There are different approaches to cope with this inaccuracy, such as the use of hybrid functionals [19], a self-interaction correction (SIC) [20], or a Hubbard-*U* correction that acts on the *d*-electrons of the TM as an effective potential (DFT+*U*) [21].
- In case that the DFT+*U* method is used to obtain a corrected total energy of a TMO, the formation energy contains an error if the total energies of the elemental reference phases were calculated by LDA or GGA, as it is generally done for the elemental phases and for compounds not containing the TM elements. The error can be systematically corrected by applying a method worked out by Jain et al. [14], where the total energies from the DFT+*U* calculations are shifted by a constant amount per TM atom. The approach is used in the MP database [18], denoted by the term *advanced corrections*, to obtain formation energies of TM-containing compounds.

This paper aims at providing a complete picture of the derivation of formation energies and phase diagrams of TMOs based on DFT+*U* and the proposed correction schemes by evaluating, discussing, and generalizing the approaches proposed in the literature. After a description of the employed methods in Section 2, we first revisit the correction of the oxygen reference energy (Section 3.1). The approach described in the literature [13] is extended by taking into account a larger set of non-TM oxides from the alkali, alkaline earth, boron, and carbon groups of the periodic table of elements in their ground state structures. The uncertainty of the resulting correction value is quantified and taken into account in the following calculations. Explicitly for Fe-containing oxides, we review in Section 3.2 the commonly used scheme [14] to correct for the error arising when comparing total energies from the DFT+*U* and DFT calculations. The underlying assumption, that the deviation between calculated and experimentally determined formation energies linearly approaches zero with the decreasing TM content, is checked by applying a general linear function and including ternary compounds with lower TM contents in the procedure. We performed the analysis for different *U* values acting on the 3*d* orbitals at Fe sites, which directly led us to a way of finding an optimal *U* value by minimizing the total mean squared deviation between the experimental and calculated formation energies for the considered compounds (Section 3.3). The widely used *U*_Fe_ value of approximately 4 eV [13,22,23,24,25,26] is confirmed, and it provides reasonable band gap predictions for the considered Fe oxides (Section 3.4).

Using the corrected formation energies, we determined the phase diagrams for LaFeO_3_, NaFeO_2_, and Li_5_FeO_4_, which are technologically promising as materials for electrodes in SOFC/SOEC devices [10,11], as positive electrodes in Na-ion batteries [27,28], and as multi-redox active cathodes in Li-ion batteries [29], respectively. In the cited literature, these compounds are described to be synthesized by solid-state reaction routes. This is a well-established technique to process ternary and higher-component oxide compounds, where oxidic precursor phases are mechanically mixed and exposed to an oxygen atmosphere of variable temperature and pressure [29]. In order to reflect this process in phase diagrams, we present these in Section 3.5 not in the conventional way with respect to elemental reservoir energies of the nongaseous components but with respect to the chemical potentials of oxidic solid precursor compounds and molecular oxygen gas.

The appendix provides a table listing experimental reference values and calculated formation energies (Appendix A), a heuristic explanation of the dependence of oxidation energies on *U* (Appendix B), a formal comparison of the method for determining an optimal *U* described in Section 3.3 and the approach using oxidation energies described by Wang et al. [13] (Appendix C), and a detailed description of the transformation of variables in the phase diagrams (Appendix D).

## 2. Methods

### 2.1. DFT(+U) Calculations of Total Energies

Total energies of elemental and compound phases were calculated using the method of density functional theory (DFT). The plane-wave-based DFT code *VASP* [30] was used for this purpose, with strict convergence criteria that ensure accurate results. A cutoff energy of 600 eV was set for the plane-wave basis functions describing the valence electrons. The partial occupancies were set according to the linear tetrahedron method with Blöchl corrections [31], except for the O_2_ molecule, for which Gaussian smearing was applied with a width of 0.05 eV. The interaction with the core electrons was modeled by projector-augmented-waves (PAW) pseudopotentials [32]. The generalized gradient approximation (GGA) of Perdew et al. [33] was chosen for the exchange-correlation (xc) functional. Electronic self-consistency loops were stopped when the energy difference between two steps was less than 10^−5^ eV, and the structures were relaxed until the minimal force component acting on an atom was below 10^−4^ eV/Å. Brillouin zone integrations were performed on grids with about 40^3^/V *k*-points, with V denoting the initial supercell volume in Å^3^. Cell volumes were optimized by total energy minimization, as implemented in *VASP*. All calculations were performed for cells with periodic boundary conditions. For the calculations of compounds containing Fe, the Hubbard-*U* correction of Dudarev et al. [34] was applied to the Fe 3*d* orbitals in order to account for the artificial self-interaction and, concomitantly, the too-weak localization of the strongly correlated *3d* electrons. Spin-polarization was taken into account, and for the following phases, the initial magnetic moments were set up according to the known ground-state spin configurations: Fe (ferromagnetic (FM)), FeO (antiferromagnetic (AFM)), Fe_2_O_3_ (AFM), Fe_3_O_4_ (ferrimagnetic), and LaFeO_3_ (AFM). All other considered iron compounds were set up in FM spin configurations.

### 2.2. Correction of the O_2_ Over-Binding

Equation (1) defines the formation energy of one formula unit of a binary oxide compound $A_{l}O_{n}$ with respect to the elemental phases of a nonoxygen component A and oxygen:

(1) $$E^{\mathrm{form}}\left(A_{l}O_{n}\right)=E^{\mathrm{total}}\left(A_{l}O_{n}\right)-\left[l\mu^{\left(0\right)}\left(A\right)+n\mu^{\left(0\right)}\left(O\right)\right].$$

$E^{\mathrm{total}}$ denotes the total energy per formula unit of the compound. The total energies per atom of the elements in their ground states are expressed by the chemical potentials $\mu^{\left(0\right)}$. Equation (1) can straightforwardly be adapted to systems containing multiple nonoxygen components, e.g., $A_{l}A_{l^{\prime }}^{\prime }$. The natural reference state of oxygen is the O_2_ molecule, with the energy $\mu^{\left(0\right)}\left(O\right)=0.5\mu^{\left(0\right)}\left(O_{2}\right)$. If the energy of an isolated O atom is set to zero, $\mu^{\left(0\right)}\left(O_{2}\right)$ is the binding energy of the molecule. Its absolute value is known to be overestimated in DFT using LDA or GGA, corresponding to a too-strong binding [17]. Wang et al. [13] proposed a method to correct this error by comparing calculated with experimentally determined values of formation energies for a series of oxides: Li_2_O and Na_2_O, MgO and CaO, Al_2_O_3_, and SiO_2_. They chose compounds without transition metals to avoid interference between the errors originating from the over-binding of O_2_ and from combining DFT with the DFT+*U* results. The method is reevaluated and discussed in this work (Section 3.1) for a larger set of oxides from the nontransition metals in the main groups I, II, III, and IV of the periodic table of elements.

### 2.3. Energy Correction Method for the Combined DFT and DFT+U Results

In addition to the O_2_ over-binding error, a systematic inaccuracy is introduced if one calculates the formation energies of oxide compounds containing a transition metal element M, e.g., binary systems $M_{m}O_{n}$ or general compounds $A_{l}M_{m}O_{n}$, by comparing the total energies of the oxide compounds calculated by DFT+*U* to the total energies of the elemental phases, which are generally determined by DFT. The application of *U* on the 3*d* orbitals of the transition-metal atoms M in the compound shifts the reference energy. Hence, the results of calculations involving M obtained by the two different methods cannot directly be compared. As described by Jain et al. [14], the value of this error can again be systematically estimated by comparing calculated to experimental formation energy values for a set of compounds $\left\{A_{l}M_{m}O_{n}\right\}$. The concept behind this approach is that the formation energy differences per atom,

(2) $$\Delta e(A_{l}M_{m}O_{n}):=\left[E_{\mathrm{DFT}/\mathrm{DFT}+U\;}^{\mathrm{form}}\left(A_{l}M_{m}O_{n}\right)-E_{\exp.}^{\mathrm{form}}\left(A_{l}M_{m}O_{n}\right)\right]/N_{\mathrm{fu}},$$

between the combined DFT and DFT+*U* results and the experimental values are expected to depend linearly on the fraction $x_{M}:=m/N_{\mathrm{fu}}$ of the transition metal M in the formula unit, which contains a total number of $N_{\mathrm{fu}}=l+m+n$ atoms.

For the case of iron, i.e., M=Fe, the binary oxide compounds FeO, Fe_2_O_3_, and Fe_3_O_4_ were taken into account in the original work [14], and their energies were calculated using a given, independently determined *U*_Fe_ value of 4 eV [13]. The data points Δe were fitted by a line through the origin, $\Delta e^{\mathrm{fit},0}\left(x_{\mathrm{Fe}}\right)=m_{0}\cdot x_{\mathrm{Fe}}$, justified by the argument that, without any transition metal atoms in the compound, and considering an O_2_ over-binding correction as described in Section 2.2, there should be zero deviation between the calculated and experimental results. This opens up the possibility to correct the DFT/DFT+*U* combination error for any iron-containing oxide compound by using the so-determined slope $m_{0}$ of the best-fit line.

In this work (Section 3.2), we compare this method to a more general approach, where the best-fit line is not forced through the origin, i.e., $\Delta e^{\mathrm{fit}}\left(x_{\mathrm{Fe}}\right)=m\cdot x_{Fe}+c$. It is shown that the line approximately approaches the origin if both binary iron oxides and representative ternary compounds are taken into account. In addition, we tested this method for five different values of *U*_Fe_ in order to discuss its generality and applicability. A *U* value is derived, for which the corrected values are, on average, as close as possible to the experimental results (Section 3.3). The oxygen correction derived in Section 3.1 was applied, and the effect of its uncertainty on the DFT/DFT+*U* correction is discussed.

### 2.4. Derivation of Phase Diagrams

The synthesis process of a material can be imagined as an exchange of elemental components and precursor compounds between reservoirs and the forming phase. Assuming thermodynamic equilibrium, the total energy of a solid phase—say $,\;A_{l}M_{m}O_{n}$—can be expressed as the stoichiometric sum of the reservoir energies per atom—namely, their chemical potentials μ. Using Equation (1), this leads, in the case of elemental reservoirs, to a formation energy:

(3) $$E^{\mathrm{form}}\left(A_{l}M_{m}O_{n}\right)=l\Delta \mu_{A}+m\Delta \mu_{M}+n\Delta \mu_{O}.$$

Here, $\Delta \mu_{i}≔\mu_{i}-\mu_{i}^{\left(0\right)}$, i.e., the chemical potentials are referenced to the energies of the elemental ground state phases. This formulation allows to relate the theoretical definition to experimental synthesis conditions, which can be described as “rich” in a component X, if $\Delta \mu_{X}$ is close to zero, and as “poor” in X, if $\Delta \mu_{X}$ has a large negative value. In the X-rich case ($\Delta \mu_{X}=0$), the elemental phase X forms in its ground state with energy $\mu_{X}^{\left(0\right)}$, imposing the constraints $\Delta \mu_{X}<0$ on the chemical potentials of all involved elements for the formation of a single compound phase.

Analogously, to prevent the formation of competing, unwanted phases from a subset of the provided components (e.g., $A_{l^{\prime }}O_{n^{\prime }}$), the stoichiometric sum of the corresponding chemical potentials must be lower than the formation energy of the wanted phase (e.g., $l^{\prime }\Delta \mu_{A}+n^{\prime }\Delta \mu_{O}<E^{\mathrm{form}}\left(A_{l^{\prime }}O_{n^{\prime }}\right)$). The phase diagram of $A_{l}M_{m}O_{n}$ is then defined by the space of chemical potentials fulfilling Equation (3) together with every possible constraint of such a form. For a ternary compound, it can be visualized by a 2-dimensional graph with two of the chemical potentials as axes and the third one sketched by contour lines or color coding. In such a plot, lines represent the constraints confining the stability region.

The chemical potential of a gaseous phase, such as O_2_, can be formally expressed as a function of the temperature and pressure of the gas [35]. If the synthesis takes place under a gas atmosphere, this links the chemical potential directly to accessible experimental processing parameters. However, it is difficult to give a quantitative interpretation of the chemical potentials of substances not provided as a gas during the synthesis beyond statements of conditions being “rich” or “poor” in the respective component.

In order to reflect the solid-state reaction process in the phase diagram, where often precursor compounds are mechanically mixed and heat-treated rather than nongaseous elemental phases, the axes need to be transformed to the chemical potentials of such compound phases and $\Delta \mu_{O}$. The formalism is explicitly demonstrated in the appendix (Appendix D) for the example of the synthesis of LaFeO_3_ from La_2_O_3_ and Fe_2_O_3_. It can be adopted to describe various processing routes for arbitrary compounds.

## 3. Results and Discussion

### 3.1. O_2_ Correction

**Figure 1:**
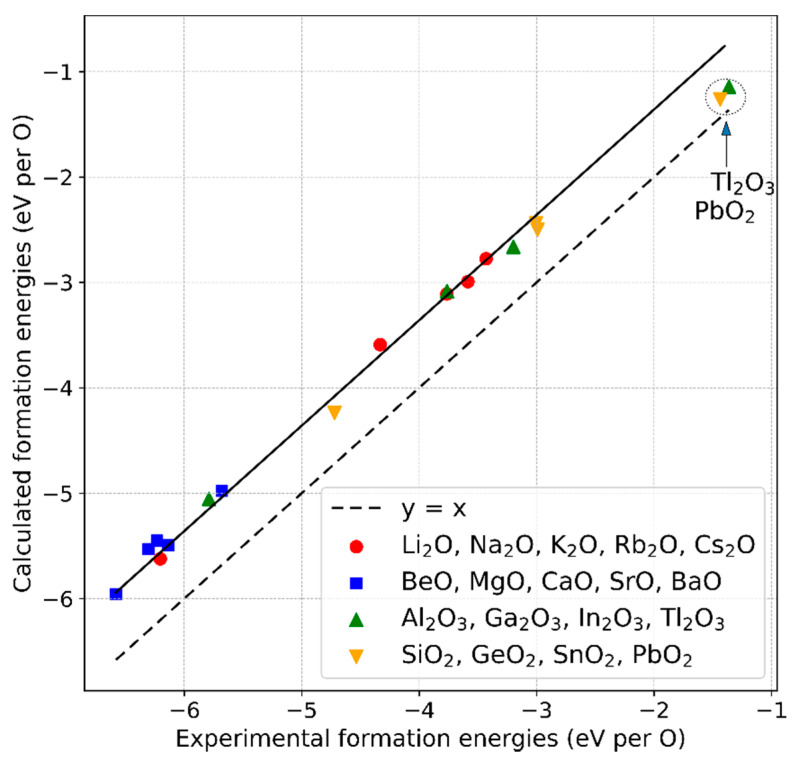
Comparison between formation energies derived experimentally (standard enthalpies of formation) and by density functional theory (DFT) calculations for a series of non-transition-metal oxides. The continuous line is a linear fit through the data points (excluding Tl_2_O_3_ and PbO_2_): y=x+b, with b= 0.64 eV/O. The dashed line is the identity line (y=x).

Using the computational DFT settings described in Section 2.1, the total energies were calculated for binary oxide compounds composed of elements from the main groups I to IV of the periodic table: $A_{2}O$ with A = Li–Cs, AO with A = Be–Ba, $A_{2}O_{3}$ with A = Al–Tl, and ${\mathrm{AO}}_{2}$ with A = Si–Pb. For those compounds, we chose the most stable structures according to experimental observations at ambient conditions (see Table A1 in Appendix A). We calculated the formation energies according to Equation (1) from the total energies of the corresponding elemental ground-state phases (the chemical potentials $\mu^{\left(0\right)}$). In Figure 1, the results are plotted against experimental values of the standard enthalpies of the formation taken from References [36,37,38,39]. With the exception of Tl_2_O_3_ and PbO_2_, the points can be represented by a line, y=x+b, which confirms the generality of the approach of Wang et al. [13], where six arbitrarily chosen compounds were taken into consideration for this analysis.

Excluding Tl_2_O_3_ and PbO_2_, a fit of the data points leads to b=0.64 eV, which can be regarded as an average energy error per O atom in the simulations. The derived b is close to the value given by Wang et al. of 0.68 eV/O [13] and to 0.7 eV/O, which is used in the (MP) database [18]. The correction can now be applied by adding b to the chemical potential of oxygen:

(4) $$\mu_{\mathrm{corrected}}^{\left(0\right)}\left(O\right):=\mu^{\left(0\right)}\left(O\right)+b.$$

Performing the correction in this way reflects that the deviation of the formation energies from the experimental values has its origin in an inaccuracy in $\mu^{\left(0\right)}\left(O\right)$ rather than in $E^{\mathrm{total}}\left(A_{l}O_{n}\right)$. However, the same results are obtained for subtracting the product nb from $E^{\mathrm{total}}$ and using the unchanged $\mu^{\left(0\right)}\left(O\right)$, as it is done in the MP approach (denoted there as “MP anion correction”).

From our results, clear trends of the data points belonging to different groups of the periodic table cannot be identified, except for a clustering of group II compounds at the lower formation energy values (blue squares) and a slight but systematic offset of the group IV compounds from the best-fit line (yellow upward triangles).

Such an offset was reported by Wang et al. for SiO_2_ [13] as well and explained by the highly covalent character of the Si–O bond. As in the case of the O_2_ molecule, the energy of such a bond is, to some extent, affected by the over-binding tendency of oxygen, leading to partial error cancellation and a formation energy of the compound closer to the experimental value. As apparent from our data, this argument can be applied for GeO_2_ and SnO_2_ as well. Mixed bonding configurations in the oxides of the heavy elements Pb and Tl could be the reason for an even stronger error cancellation in the case of the formation energies of PbO_2_ and Tl_2_O_3_. As an alternative or complementary approach to explain the deviation of these points from the best-fit line, we calculated the energies of the compounds PbO_2_ and Tl_2_O_3_ and reference phases Pb and Tl by taking into account spin-orbit coupling. This led to energy shifts of the order of 0.1 eV/O in the direction of the best-fit line.

The mean deviation of all considered data points from the best-fit line is ±0.09 eV/O, and the cited values used by other research groups are within this range as well. This range of b values has to be taken into account in further calculations using the O_2_ correction value. Next to numerical fluctuations, which we reduced as much as possible by applying strict convergence criteria, uncertainties in the experimental data may contribute to the uncertainty of the correction value as well.

Note that the measurements are carried out at ambient conditions, i.e., at room temperature and atmospheric pressure. On the other hand, the calculated formation energies are derived at zero temperature and pressure. An energy contribution $\Delta \mu \left(O_{2};T,p\right)$ can, in principle, be added to the ground state chemical potential of the oxygen in Equation (1). It can be formally derived from statistical mechanics considering the entropy effects from the different degrees of freedom of the O_2_ molecule [35]. However, such a correction must not be additionally included in the method described above, since it is already compensated for by the shift b, which encompasses both the O_2_ over-binding error inherent in DFT, as well as energy contributions due to differing external conditions. Analogously, this argumentation is valid for energy shifts Δμ(A;T,p) of the nonoxygen components and $\Delta E^{\mathrm{total}}\left(A_{l}O_{n};T,p\right)$ of the compound of interest. In order to quantify separately the effects from O_2_ over-binding and different external conditions, all of these contributions need to be explicitly calculated, e.g., by considering volume changes with the temperature and applying an equation of state or by performing thermodynamic integrations of heat capacities [15].

An alternative way to deal with the effect of O_2_ over-binding in DFT was recently reported by Gautam and Carter [40]. They applied the strongly constrained and appropriately normed (*SCAN*) approximation for the exchange-correlation functional and obtained formation energies of main group oxide compounds, which agreed quite well with the experimental values without the need to perform any post-processing corrections. With our study, though, we aimed to evaluate and discuss common, widely applied DFT methods, which are implemented in many of the available codes and which, for example, were used to generate large sets of data as available in the MP database [18].

### 3.2. Energy Correction for the Combined DFT and DFT+U Results

**Figure 2:**
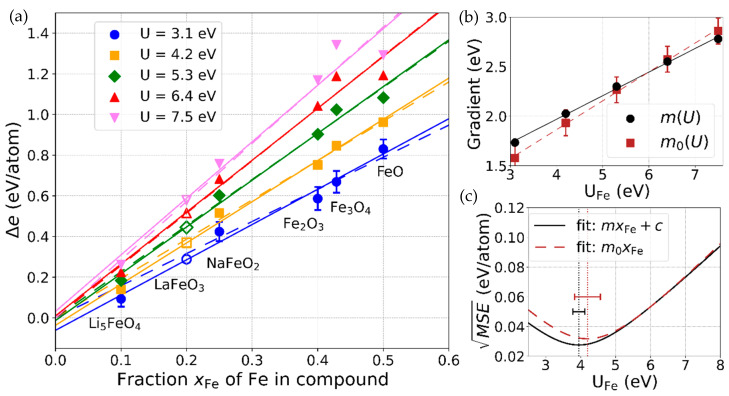
(**a**)**:** Comparison of the calculated data for different *U* values with the experimental data. Symbols: energy differences Δe between formation energies calculated with DFT and DFT+*U* and experimental values for different compounds containing Fe and for different values of *U*_Fe_. The error bars shown exemplarily for the values for *U*_Fe_ = 3.1 eV originate from the uncertainty in the oxygen correction value b, described in Section 3.1. They are not to be understood in a statistical way but meant as upper and lower limits between which all of the data points consistently shift if b is varied between −Δb and +Δb. Data points represented by filled symbols were fitted by linear functions $\Delta e_{U}^{\mathrm{fit}}\left(x_{\mathrm{Fe}}\right)=m\left(U\right)x_{\mathrm{Fe}}+c\left(U\right)$ (solid lines) and $\Delta e_{U}^{\mathrm{fit},\;0}\left(x_{\mathrm{Fe}}\right)=m_{0}\left(U\right)x_{\mathrm{Fe}}$ (dashed lines). The open symbols for LaFeO_3_ were not included in the fitting. (**b**): Slopes m(U) and $m_{0}\left(U\right)$. Again, the error bars depict the upper and lower limits of the values. They result from the limits of the data points in the graph in the left panel. (**c**): Square root of the mean square errors (MSE) between the fit function $\Delta e_{U}^{\mathrm{fit}}\left(x_{\mathrm{Fe}}\right)$ ($\Delta e_{U}^{\mathrm{fit},0}\left(x_{\mathrm{Fe}}\right)$) and the data points Δe. The *U* values leading to the MSE minima are displayed by dashed vertical lines at 3.95 eV ($\Delta e_{U}^{\mathrm{fit}}$) and 4.20 eV ($\Delta e_{U}^{\mathrm{fit},0}$), together with bars encompassing again the range of values that result when the oxygen correction is consistently varied between b−Δb and b+Δb.

Figure 2a depicts the differences Δe defined in Equation (2) between the formation energies per atom derived by using the results from the DFT and DFT+*U* calculations and the corresponding experimental standard enthalpies of the formation of the binary compounds FeO, Fe_2_O_3_, and Fe_3_O_4_ and the ternary compounds Li_5_FeO_4_ and NaFeO_2_. The phases were set up in their experimentally observed ground-state structures (see Table A1 in Appendix A).

The Δe values are plotted against $x_{\mathrm{Fe}}$, the fraction of Fe atoms in the compound. The ternary compounds were included to add more data points for the lower $x_{\mathrm{Fe}}$ values. They were chosen based on the availability of the experimental data and such that they do not contain any further transition metal elements. The data points Δe were fitted by a straight line

(5) $$\Delta e_{U}^{\mathrm{fit}}\left(x_{\mathrm{Fe}}\right)=m_{U}\cdot x_{\mathrm{Fe}}+c_{U}$$

for each of the five considered *U* values for Fe between 3.1 eV and 7.5 eV. A linear fit is well-justified for most of the data points, except for those belonging to Fe_3_O_4_ and FeO obtained with the higher *U* values. The lines $\Delta e_{U}^{\mathrm{fit}}\left(x_{\mathrm{Fe}}\right)$ have slopes $m_{U}$ and y-intercepts $c_{U}$, which themselves exhibit linear trends as a function of *U*: $m_{U}=m\left(U\right)\approx 0.24\cdot U+\left(1.02\mp 0.04\right)$ eV and $c_{U}=c\left(U\right)\approx 0.02\cdot U-\left(0.13\pm 0.04\right)$ eV. m(U) is displayed in Figure 2b. The given ranges of the y-intercepts of m(U) and c(U) stem from the uncertainty limits ±Δb in the O_2_ over-binding correction described in the previous section. By changing b within these limits, the energy differences Δe shown in Figure 2a are consistently shifted to higher or lower values between the limits, which, for the sake of clarity, are exemplarily depicted by error bars only for the data points corresponding to *U* = 3.1 eV. The presented numbers determining $\Delta e_{U}^{\mathrm{fit}}\left(x_{\mathrm{Fe}}\right)$ are, of course, sensitive to the incorporation of additional ternary Fe-containing compounds in the analysis. However, this effect is of minor influence, as long as the data points of additional compounds do not deviate more strongly from the lines than those already considered.

$\Delta e_{U}^{\mathrm{fit}}\left(x_{\mathrm{Fe}}\right)$ shifts the calculated formation energy per atom of an arbitrary oxide compound with a fraction of $x_{\mathrm{Fe}}$ Fe atoms closer to its experimental value. Following the definition of the formation energy (Equation (1)), this correction can be formally applied by changing the total energies calculated with DFT+*U* at a given *U*, according to:

(6) $$E_{U,\;\mathrm{corr}}^{\mathrm{total}}\left(A_{l}{\mathrm{Fe}}_{m}O_{n}\right):=E_{U}^{\mathrm{total}}\left(A_{l}{\mathrm{Fe}}_{m}O_{n}\right)-N_{\mathrm{fu}}\Delta e_{U}^{\mathrm{fit}}\left(x_{\mathrm{Fe}}\right).$$

As a proof of principle, correcting the energies $E_{U}^{\mathrm{total}}\left({\mathrm{LaFeO}}_{3}\right)$ in this manner leads to formation energies very close to the experimental values. This can be seen in Figure 2a, where the open symbols corresponding to LaFeO_3_, which were not included in the fitting processes, closely match the values of $\Delta e_{U}^{\mathrm{fit}}\left(x_{\mathrm{Fe}}=0.2\right)$ for all considered *U* values.

In order to compare this generalized approach for deriving the correction energy arising in the combined DFT and DFT+*U* calculations to the method described by Jain et al. [14], we repeated the whole analysis using best-fit lines through the origin,

(7) $$\Delta e_{U}^{\mathrm{fit},\;0}\left(x_{\mathrm{Fe}}\right)=m_{0}\left(U\right)\cdot x_{\mathrm{Fe}},$$

marked by the dashed lines in Figure 2a. For the higher *U* values, the lines hardly deviate from the lines $\Delta e_{U}^{\mathrm{fit}}\left(x_{\mathrm{Fe}}\right)$. The slopes, which are the only correction parameters in this approach, are derived as $m_{0}\left(U\right)=0.29\cdot U+\left(0.70\mp 0.13\right)$ eV (depicted in the Figure 2b). Their range of uncertainty that again originates in the uncertainty limits ±Δb of the O_2_ over-binding correction, is enlarged, as compared to the general method.

### 3.3. Determination of U

The data points Δe and, therefore, the deviations $\delta :=\Delta e-\Delta e^{\mathrm{fit}}$, follow linear trends with *U* for each of the five iron compounds included in the fitting procedure. Using the corresponding fit parameters, the average of the squared deviations, known as the mean squared error MSE, can be obtained as a function of *U*:

(8) $$MSE\left(U\right)=\frac{1}{5}\sum_{i=1}^{5}{\left[\delta \left(x_{\mathrm{Fe}}^{\left(i\right)},U\right)\right]}^{2}.$$

The square root of this expression generally serves as a measure for the quality of the fits and, specifically, here for the quality of the correction scheme. It is displayed in the Figure 2c for both types of fitting discussed in Section 3.2. A difference is visible only for *U* values below 4.5 eV. Minimization leads to *U*_Fe_ = (3.95 ± 0.17) eV and *U*_Fe_ = (4.20 ± 0.37) eV for the general fits and the fits through the origin, respectively. The given ranges depicted by bars originate again from the uncertainty limits of the oxygen correction value. Around these minima, the application of either of the two correction schemes reproduces the experimental formation energies on average with an accuracy of about 0.03 eV per atom. The general fit leads to slightly more accurate results, but this difference can be regarded as insignificant, considering the uncertainties in the approaches.

A *U* value for the 3*d*-electrons of Fe of 4.0 ± 0.1 eV was also derived by Wang et al. [13] following a different but related approach. The authors calculated oxidation reaction energies $E^{\left(r\right)}$ between the binary iron oxides FeO, Fe_2_O_3_, and Fe_3_O_4_. No elemental Fe phase is involved in these reactions. Therefore, $E^{\left(r\right)}$ could be determined by balancing only the uncorrected total energies obtained by DFT+*U* and the previously corrected chemical potential of oxygen. The change of $E^{\left(r\right)}$ with *U*, which can be understood heuristically (see Appendix B), led to an apparently optimal *U* value where the results for $E^{\left(r\right)}$ best matched the experimental reaction energies. $E^{\left(r\right)}$ depends linearly on the formation energies of the involved compounds. Since these values also enter the method described in this section, the approach of Wang et al. may be interpreted as being formally equivalent to the method described above if only the binary oxides were included in the fitting. This is, however, not the case, as is shown in detail in Appendix C.

A *U*_Fe_ value of approximately 4 eV is widely reported in the literature to lead to band gaps and reaction energies of iron oxides in good agreement with experimentally derived values [13,22,23,24,25,26]. It has to be noted that, in all of the cited studies, as in this work, the DFT code *VASP* was used together with the standard set of PAW pseudopotentials provided in the *VASP* package. In contrast, Xu et al. [41] reported optimal values of *U*_Fe_ for the iron oxides, which were derived with the DFT code Quantum ESPRESSO (QE) [42] by the linear response method [43]. With *U*_Fe_ between 3.47 eV and 4.10 eV, the values they obtained using pseudopotentials from the original QE library were in acceptable agreement with the *VASP* values. However, considerably different *U*_Fe_ values between 5.21 eV and 6.07 eV resulted from the application of QE with a different set of pseudopotentials [41], namely those from the GBRV high-throughput library [44]. This shows that one has to be careful in proposing and adopting universal element dependent *U* values, since they can strongly depend on the choice of the pseudopotential and, additionally, may differ between DFT codes due to different implementations of the Hubbard-*U* correction.

### 3.4. Band Gaps of the Iron Compounds

**Figure 3:**
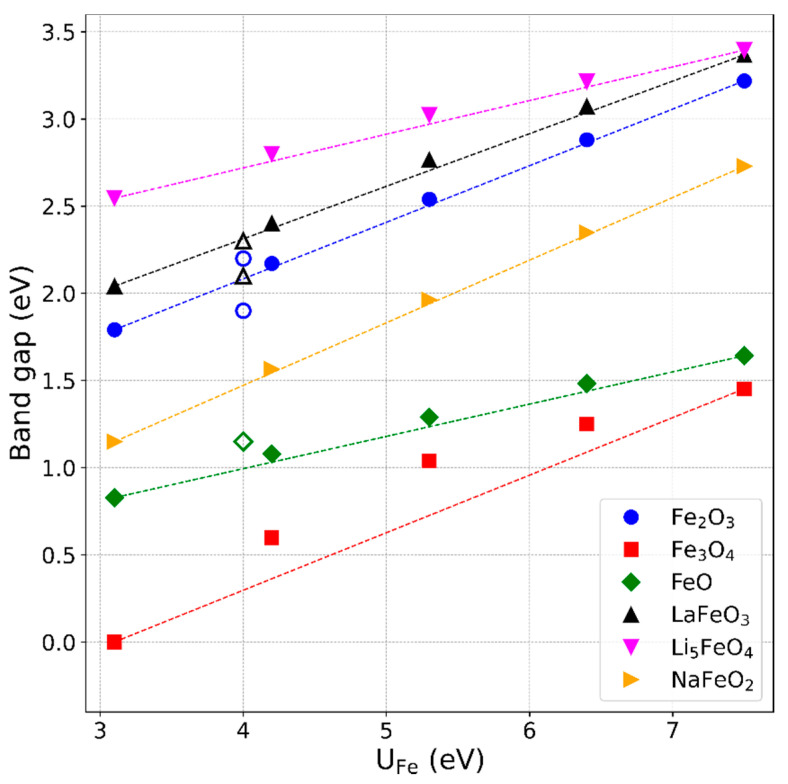
Calculated band gap energies as functions of *U*. Filled symbols: band gaps of iron-oxide compounds calculated with DFT+*U* for different *U* values. The lines connect the two outer data points, indicating a more-or-less pronounced upwards shift of the values around *U* ≈ 5.3 eV. Open symbols, exemplarily displayed at *U =* 4 eV, represent experimental band gap values of the respective compounds [45,46,47,48,49].

The usual approximations of DFT, LDA, or GGA insufficiently describe the correlated electrons in 3*d*-orbitals of transition metals, which, together with the oxygen 2*p*-orbitals, form the valence band edge in transition metal oxide compounds. This leads to incorrect, generally underestimated, band gaps, which can be opened up by applying *U* to localize the 3*d*-orbitals, making the electronic structure less “metal-like”. Accordingly, an optimal *U* value can be found by tuning *U* until the experimental band gap value is reproduced in the band-structure calculations [22]. To compare this approach to the one described in Section 3.3, we calculated the band gaps of the considered compounds for the different *U* values of iron. As shown in Figure 3, the band gaps exhibit the expected opening with increasing *U*_Fe_, following, in most cases, almost linear trends. For Fe_3_O_4_, a distinct bend of the line around *U* ≈5.3 eV is apparent. This is a significant feature that is visible, albeit much less pronounced, for the other compounds as well. Very strict convergence criteria were applied to ensure that this effect is not an artifact of numerical fluctuations but, rather, a characteristic feature of the DFT+*U* approach. On a small scale, such a bending was also observed in the total energies and the quantities deduced from them. This can be seen, for example, by closely examining the deviations of the data points from the lines in Figure 2b. However, this is considered as insignificant in the approximate analysis conducted there.

The following experimental band gap energy data were taken from the literature: for Fe_2_O_3_ 1.9 eV [45] and 2.2 eV [46], for FeO 1.15 eV [47], and for LaFeO_3_ 2.1 eV [48] and 2.3 eV [49]. These data lie within 0.2 eV around the calculated values in the region of the previously optimized value *U* ≈4 eV. The data are marked by the open symbols in Figure 3. Fe_3_O_4_ is known to be electronically conductive in the cubic state, so there should be no band gap, which is reproduced by the DFT+*U* calculations for *U* below about 3 eV. A small band gap of 0.07 eV is reported only for the monoclinically distorted structure forming below the Verwey transition temperature [50]. The complex electronic structure of Fe_3_O_4_ of mixed octahedrally coordinated Fe^3+^ and tetrahedrally coordinated Fe^2+^ ions cannot be sufficiently described by the same, single *U* value that is applied for the octahedrally coordinated Fe ions in the same trivalent charge state in the other compounds. For Li_5_FeO_4_ and NaFeO_2_, no experimental band gap values are reported, to the best of our knowledge. Hoang et al. [51] derived a band gap of 4.4 eV for Li_5_FeO_4_ using hybrid-functional DFT calculations with nonoptimized mixing or screening parameters. This value is considerably larger than the DFT+*U* results obtained here. For NaFeO_2_, only one DFT+*U* calculation was found in the literature [52] with a band gap value of 1.5 eV for *U*_Fe_ = 4 eV, in agreement with our results.

### 3.5. Phase Diagrams of LaFeO_3_, Li_5_FeO_4_, and NaFeO_2_

In order to derive the phase diagrams for the ternary oxide compounds LaFeO_3_, Li_5_FeO_4_, and NaFeO_2_, we calculated ground-state formation energies for a set of binary oxides and ternary iron oxides containing the elements La, Li, or Na, respectively. A *U* value for Fe of 4 eV was applied, and the corrections determined in Section 3.2 and Section 3.3 were adopted. The choice of compounds was based on phases listed in the MP database [18], with energies close to the convex hull. The way the phase diagrams are presented in the following reflects the solid-state processing routes that were reported for the considered materials.

#### 3.5.1. LaFeO_3_

LaFeO_3_ was experimentally synthesized by a mechanical mixing treatment of La_2_O_3_ and Fe_2_O_3_ powders and calcination under an oxygen atmosphere [53,54], according to:

$${\mathrm{La}}_{2}O_{3}\;+\;{\mathrm{Fe}}_{2}O_{3}\;⟶\;2\;\mathrm{La}\mathrm{Fe}O_{3}.$$

**Figure 4:**
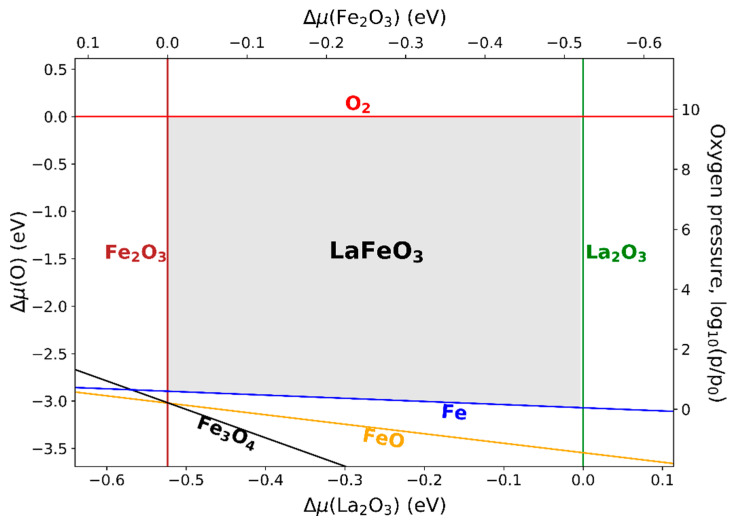
Phase diagram of LaFeO_3_ with respect to the chemical potentials of the oxide components La_2_O_3_ (Fe_2_O_3_) and oxygen. At a given temperature, which was chosen as 1400 K, in this case, $\Delta \mu_{O}$ can be expressed by the pressure of the oxygen gas (right vertical axis, logarithmic scaling in units of $p_{0}\;$= 1 atm). The lines mark the transitions to the denoted competing phases.

The phase diagram with variables $\Delta \mu \left({\mathrm{La}}_{2}O_{3}\right)$ ($\Delta \mu \left({\mathrm{Fe}}_{2}O_{3}\right)$) and Δμ(O) is shown in Figure 4, with an alternative *y*-axis assigned to the pressure of the oxygen gas at a temperature of 1400 K. In addition to the binary iron oxides and La_2_O_3_ (space group $Ia\bar{3}$), the phase La_3_FeO_6_ ($Cmc2_{1}$) was taken into account. However, it turned out that the latter is not relevant for the phase diagram of LaFeO_3_, since the corresponding phase separation line lies outside of the displayed region.

Since the stability region is horizontally confined by the precursor compounds of the reaction, the formation of an intermediate phase during the synthesis of LaFeO_3_ is not expected. Molecular oxygen and metallic Fe can form at very high and at very low gas pressures, respectively. Lines corresponding to metallic La, FeO, or Fe_3_O_4_ do not cross the stability region, indicating that no thermodynamic condition can be realized under which LaFeO_3_ would be in equilibrium with either of these phases. The functional dependencies between $\Delta \mu_{\mathrm{La}}$, $\Delta \mu_{\mathrm{Fe}}$, $\Delta \mu_{O}$, $\Delta \mu_{{\mathrm{La}}_{2}O_{3}}$, and $\Delta \mu_{{\mathrm{Fe}}_{2}O_{3}}$ (see Appendix D) can be applied to determine the chemical potentials of the metallic elements at each point in the phase diagram, which are, e.g., needed for calculating the point defect formation energies involving these elements. Heifets et al. [55] calculated and discussed the phase diagrams of LaFeO_3_ with respect to the elemental chemical potentials $\Delta \mu_{\mathrm{La}}$ and $\Delta \mu_{\mathrm{Fe}}$. The phase diagram that is derived there using the experimental input data shows chemical potentials $\Delta \mu_{O}$ between −3.2 and −2.8 eV along the $\Delta \mu_{\mathrm{Fe}}$= 0 line in agreement with our results in Figure 4. This is expected, since the calculated formation energies we used for deriving the phase diagram are close to the experimental values because of the applied correction procedure.

Although a representation of the phase diagram in the form of Figure 4 is closer to the reality of the actual synthesis process than a representation with respect to elemental reservoir energies [56,57,58,59], it remains a difficulty of adjusting and quantifying or, in general, interpreting $\Delta \mu_{{\mathrm{La}}_{2}O_{3}}$ and $\Delta \mu_{{\mathrm{Fe}}_{2}O_{3}}$ in an experimental setup. In analogy to a gas phase, “rich” conditions correspond to a high chemical reactivity, which can, for example, be realized by a high density of reactive surfaces, i.e., small and densely packed grains.

#### 3.5.2. Li_5_FeO_4_

The experimental synthesis of Li_5_FeO_4_ was achieved by tempering a powder mixture of Li_2_O and elemental Fe at 1000 °C and a low pressure of about 1 mPa [60], following the reaction:

$$4\;{\mathrm{Li}}_{2}O\;+\;\mathrm{Fe}\;\to \;{\mathrm{Li}}_{5}{\mathrm{FeO}}_{4}\;+\;3\;\mathrm{Li}.$$

**Figure 5:**
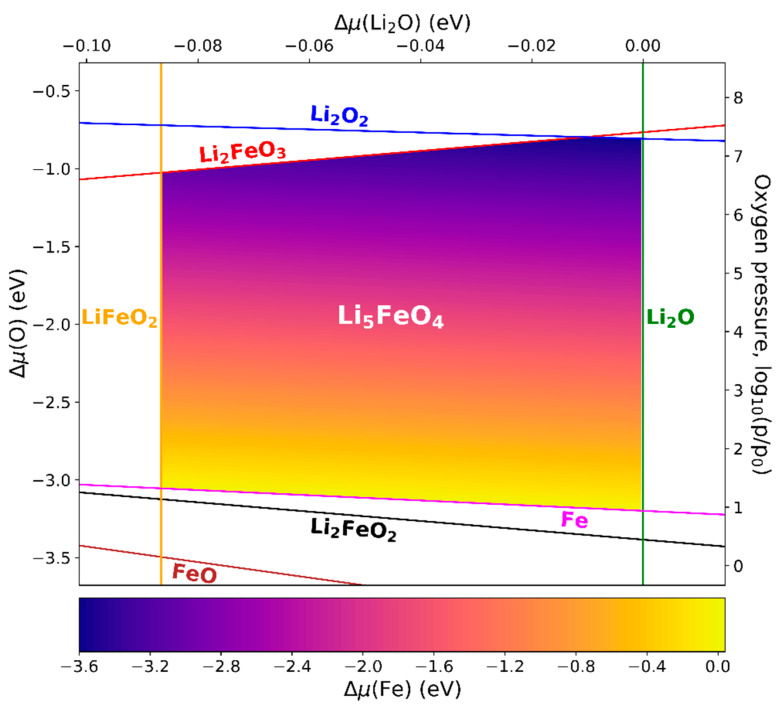
Phase diagram of Li_5_FeO_4_ with respect to the chemical potentials of Li_2_O and oxygen. The vertical axis on the right is assigned to the corresponding oxygen gas pressure at a temperature of 1300 K ($p_{0}\;$ = 1 atm). The chemical potential of Fe is visualized by color coding. The lines mark the transitions to the denoted competing phases.

The corresponding phase diagram is shown in Figure 5, with variables $\Delta \mu \left({\mathrm{Li}}_{2}O\right)$ and $p\left(O_{2}\right)$ for T=1300 K. Since Fe is involved in the reaction, its chemical potential is of relevance as well. This energy of the second reaction partner cannot be represented by an alternative horizontal axis, as in the case of LaFeO_3_ (see Figure 4), due to the existence of an additional species (Li) in the reaction (see detailed explanation in Appendix D). Instead, Δμ(Fe) was made visible via color coding in the stability region. Next to Li_2_O (space group $Fm\bar{3}m$) and the binary iron oxides, the phases taken into account were Li_2_O_2_ ($P6_{3}mmc$), Li_2_FeO_2_ (Immm), Li_2_FeO_3_ (C2), and LiFeO_2_ ($Fd\bar{3}m$).

Based on the scale of the horizontal axis, Li_5_FeO_4_ can be expected to form only under very rich Li_2_O conditions. The experimentally applied pressure of $10^{-8}$ atm corresponds to rather rich Fe conditions as well. During the vaporization of Li_5_FeO_4_, the precipitation of solid LiFeO_2_ was observed [60], accompanied by gaseous Li and O_2_. This is in agreement with the phase diagram, where LiFeO_2_ terminates the narrow stability region on one side. The Li gas cannot solidify, since the corresponding line (Δμ(Li)=0) lies far outside the Li_5_FeO_4_ region at unreasonably low oxygen gas pressures below $10^{-35}$ atm. In contrast to the situation of LaFeO_3_, the phase diagram indicates no equilibrium between Li_5_FeO_4_ and a binary iron oxide. For example, the line $\Delta \mu \left({\mathrm{Fe}}_{2}O_{3}\right)$ = 0, which is parallel to $\Delta \mu \left({\mathrm{LiFeO}}_{2}\right)$ = 0, is located at $\Delta \mu \left({\mathrm{Li}}_{2}O\right)$ = −0.18 eV outside of the displayed region. This impedes a direct solid-state route, such as Li_2_O + Fe_2_O_3_ → 2 Li_5_FeO_4_, which, at rich Fe_2_O_3_ conditions, would lead to the formation of LiFeO_2_ instead.

#### 3.5.3. NaFeO_2_

NaFeO_2_ was experimentally synthesized by milling the precursor compounds Na_2_O_2_ (sodium peroxide) and Fe_3_O_4_ and exposing the mixture to a temperature of about 900 K [27,28]. Due to stoichiometric constraints, oxygen has to be released during this reaction:

$$3\;{\mathrm{Na}}_{2}O_{2}\;+\;2\;{\mathrm{Fe}}_{3}O_{4}\;\to \;6\;{\mathrm{NaFeO}}_{2}\;+\;O_{2}.$$

**Figure 6:**
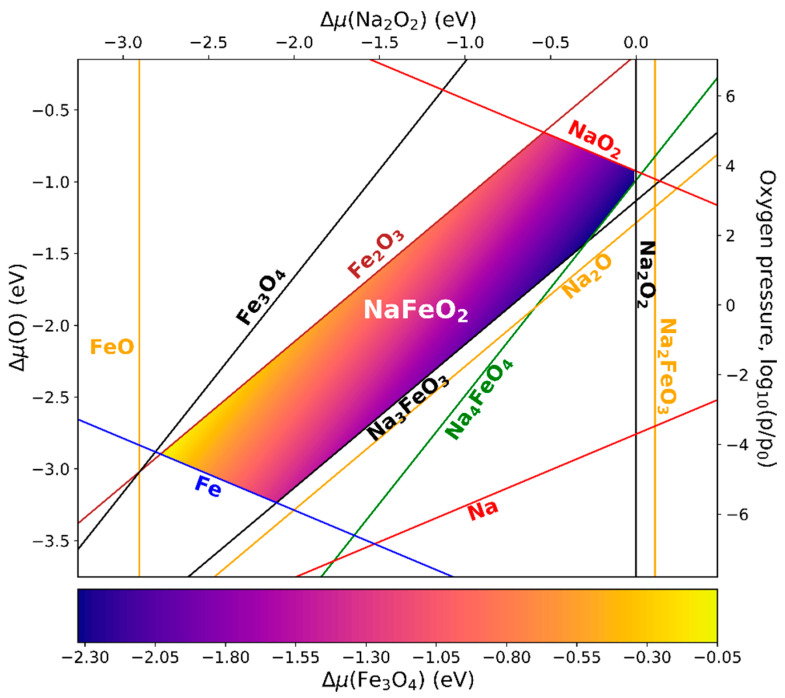
Phase diagram of NaFeO_2_ with respect to the chemical potentials of Na_2_O_2_ and oxygen. The vertical axis on the right is assigned to the corresponding oxygen gas pressure at a temperature of 900 K ($p_{0}\;$ = 1 atm). The chemical potential of Fe_3_O_4_ is visualized by color coding. The lines mark the transitions to the denoted secondary phases.

The phase diagram is presented with variables $\Delta \mu \left({\mathrm{Na}}_{2}O_{2}\right)$, Δμ(O), and $p\left(O_{2}\right)$ for $T\left(O_{2}\right)$ = 900 K in Figure 6, with the chemical potential of Fe_3_O_4_ being depicted by a color code. Next to Na_2_O_2_ (space group $P\bar{6}2m$) and the binary iron oxides, the phases taken into account were Na_2_O ($Fm\bar{3}m$), NaO_2_ (Pnnm), Na_2_FeO_3_ ($P\bar{1}$), Na_3_FeO_3_ ($P2_{1}/c$), Na_4_FeO_4_ ($P\bar{1}$), and NaFeO_2_ ($R\bar{3}m$). Further phases consisting of Na, Fe, and O turned out to be irrelevant for the stability discussion of NaFeO_2_.

Under Fe_3_O_4_-rich conditions, NaFeO_2_ only forms at low pressures and Na_2_O_2_-poor conditions (lower left part of the stability region). If the atmospheric pressure is too high, the oxidation of Fe_3_O_4_ to Fe_2_O_3_ can occur, and if it is too low, a reduction to metallic Fe is expected. Under Na_2_O_2_-rich conditions (upper right part of the stability region), NaFeO_2_ only forms at high oxygen pressures and Fe_3_O_4_-poor conditions, and the phases NaO_2_ and Na_4_FeO_4_ are likely to emerge. Based on the phase diagram, two alternatively plausible processing routes can be proposed: Na_3_FeO_3_ + Fe_2_O_3_ → 3 NaFeO_2_ and Na_2_O + Fe_2_O_3_ → 2 NaFeO_2_. The latter does not include the complication of preliminarily having to synthesize Na_3_FeO_3_, but it can only be realized under not-too-rich Na_2_O conditions. A reaction of metallic Fe with sodium superoxide (NaO_2_) appears possible as well, but due to the high reactivity of NaO_2_ with water and a costly production process, this route is presumably inexpedient from a practical point of view.

## 4. Summary and Conclusions

In the first part of this work, we compiled, reconsidered, and reevaluated a set of correction methods previously described in the literature and applied in a frequently accessed materials database (Materials Project, MP), which deals with inaccuracies of the usual LDA or GGA calculations of DFT in deriving accurate formation energies of transition metal oxide compounds. Common to these methods is the incorporation of physically justified and generally applicable parameters for systematic error corrections, which can be tuned to minimize an average deviation of computed and experimentally determined energies for a variety of compounds. We come to the following conclusions:

- The method to correct the formation energy error due to the over-binding of the O_2_ molecule remains valid if a larger set of non-transition metal oxide compounds from the groups I to IV of the periodic table is considered instead of the previously chosen smaller subset. The magnitude of the energy correction we derived (0.64 eV) agrees within 0.1 eV with the reported values, which reflects the uncertainty range we determined for the approach. Experimental enthalpies of the formations for the considered compounds can be reasonably reproduced within 0% to 5%, except for Tl_2_O_3_ and PbO_2_, for which the values deviate more. A possible explanation is given.
- For the binary iron oxide compounds, we confirmed that it is well-justified to correct the error arising in combining the results of DFT and DFT+*U* calculations by adding a value proportional to the Fe content to the formation energy. While it was originally derived considering only the binary oxides for a fixed *U* value for Fe, we strengthened and generalized the scheme by (a) taking into account ternary compounds, (b) not a priori constraining the correction value to be zero for hypothetical compounds without Fe, and (c) considering different values of *U*.
- Our *U*-dependent correction value offers a new possibility to determine an optimal *U* value for Fe for which the experimental formation energies are reproduced best. With this approach, we confirmed the frequently used value of *U* ≈ 4 eV, which additionally turned out to reproduce experimental band gaps of the considered compounds within 0.3 eV.

In the second part of this work, by applying the above-mentioned correction schemes, we calculated the formation energies for a set of binary oxide and ternary iron oxide compounds containing La, Li, or Na in order to derive the phase diagrams for LaFeO_3_, Li_5_FeO_4_, and NaFeO_2_ with respect to the reservoir energies (chemical potentials) of elements and/or precursor compounds. Our representation of the phase diagrams corresponds closely to actual solid-state synthesis routes reported in the experimental literature. This allows for motivating the experimental adjustments of pressures, for predicting the phase transformations upon changing the synthesis conditions, and for explaining the phases found after vaporization, which are demonstrated for the described compounds. In addition, alternative synthesis routes can be proposed and compared.

## Figures and Tables

**Figure A1:**
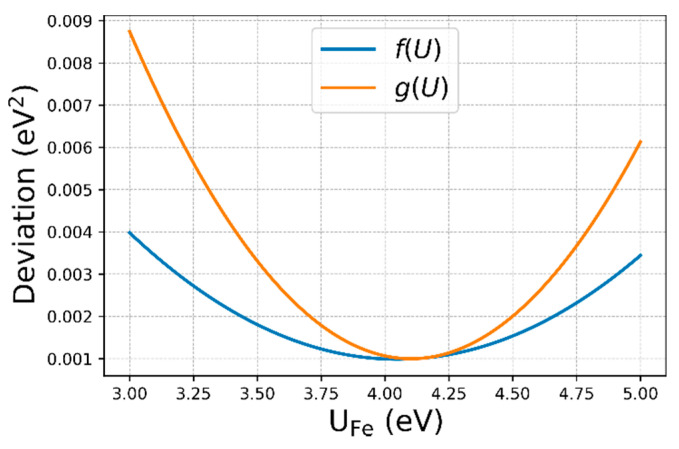
Deviation functions of formation energy differences between the calculated and experimental values from the linear fit (f(U)) and of calculated oxidation energies from the corresponding experimental values (g(U)).

## Appendix A: List of Calculated and Experimental Formation Energies

**Table A1:** Formation energies of oxide compounds in their ground state crystal structure (specified by the space group (SG) in Hermann-Mauguin notation) with respect to the elemental phases in eV per formula unit. Experimental standard enthalpies of the formation ($E_{\exp.}^{\mathrm{form}}$) are taken from References [38] (Rb_2_O and Cs_2_O); (Ga_2_O_3_, In_2_O_3_, GeO_2_, SnO_2_, and PbO_2_) [37]; (Tl_2_O_3_) [39]; (LaFeO_3_) [53]; (Li_5_FeO_4_) [60]; (NaFeO_2_) [61]; and [36] (all other compounds). Calculated formation energies from DFT (non-TM oxides) and DFT+*U* for *U*_Fe_ = 4 eV (TM oxides) are given without any corrections ($E_{\mathrm{uncorr}.}^{\mathrm{form}}$), considering the oxygen over-binding corrections ($E_{\mathrm{ox}}^{\mathrm{form}}$) and considering the oxygen over-binding together with DFT/DFT+U combination corrections ($E_{\mathrm{ox}+U}^{\mathrm{form}}$). δ denotes the absolute value of the relative deviation of the calculated and totally corrected value from the experimental value.

Compound	SG	$E_{\exp.}^{\mathrm{form}}$	$E_{\mathrm{uncorr}.}^{\mathrm{form}}$	$E_{\mathrm{ox}}^{\mathrm{form}}$	$E_{\mathrm{ox}+U}^{\mathrm{form}}$	δ (%)
**Non-TM oxides**						
Li_2_O	$Fm\bar{3}m$	−6.21	−5.62	−6.26	-	0.8
Na_2_O	$Fm\bar{3}m$	−4.33	−3.59	−4.23	-	2.3
K_2_O	$Fm\bar{3}m$	−3.76	−3.11	−3.75	-	0.3
Rb_2_O	$R\bar{3}m$	−3.43	−2.77	−3.42	-	0.3
Cs_2_O	$R\bar{3}m$	−3.59	−2.99	−3.63	-	1.1
BeO	$P6_{3}mc$	−6.31	−5.53	−6.17	-	2.2
MgO	$Fm\bar{3}m$	−6.23	−5.45	−6.09	-	2.2
CaO	$Fm\bar{3}m$	−6.58	−5.96	−6.60	-	0.3
SrO	$Fm\bar{3}m$	−6.14	−5.49	−6.14	-	0.0
BaO	$Fm\bar{3}m$	−5.68	−4.98	−5.62	-	1.1
Al_2_O_3_	$R\bar{3}c$	−17.37	−15.16	−17.08	-	1.7
Ga_2_O_3_	C2/m	−11.29	−9.25	−11.17	-	1.1
In_2_O_3_	$Ia\bar{3}$	−9.60	−7.99	−9.91	-	3.2
Tl_2_O_3_	$Ia\bar{3}$	−4.09	−3.43	−5.35	-	30.8
SiO_2_	$I\bar{4}2d$	−9.44	−8.48	−9.76	-	3.4
GeO_2_	$P3_{1}21$	−6.01	−4.88	−6.16	-	2.5
SnO_2_	$P4_{2}mnm$	−5.99	−5.00	−6.28	-	4.8
PbO_2_	$P4_{2}mnm$	−2.88	−2.52	−3.80	-	31.9
**TM oxides**						
FeO	$Fd\bar{3}m$	−2.82	−0.31	−0.95	−2.83	0.4
Fe_2_O_3_	$R\bar{3}c$	−8.56	−3.04	−4.96	−8.69	1.5
Fe_3_O_4_	$Fd\bar{3}m$	−11.62	−3.35	−5.92	−11.53	0.8
LaFeO_3_	Pnma	−14.24	−10.59	−12.51	−14.27	0.2
Li_5_FeO_4_	Pbca	−20.21	−16.34	−18.91	−20.45	1.2
NaFeO_2_	$R\bar{3}m$	−7.23	−3.96	−5.24	−7.05	2.5

## Appendix B: Dependence of Oxidation Energies on *U*

In order to understand the dependence of the calculated oxidation reaction energy values on *U*, we consider, as an example, the reaction between FeO and Fe_2_O_3_, where Fe changes its oxidation state from 2+ to 3+:

(A1) $$2\;\mathrm{FeO}\;+\;0.5\;O_{2}\;\to \;{\mathrm{Fe}}_{2}O_{3}.$$

The corresponding reaction energy per formula unit can be expressed as:

(A2) $$E^{\left(r\right)}=E^{\mathrm{total}}\left({\mathrm{Fe}}_{2}O_{3}\right)-\left[2E^{\mathrm{total}}\left(\mathrm{FeO}\right)+0.5\mu \left(O_{2}\right)\right].$$

The application of *U* changes the total energies. We define

(A3) $$\delta_{{\mathrm{Fe}}^{3+}}:=\frac{1}{2}\left[E_{U_{>}}^{\mathrm{total}}\left({\mathrm{Fe}}_{2}O_{3}\right)-E_{U_{<}}^{\mathrm{total}}\left({\mathrm{Fe}}_{2}O_{3}\right)\right],$$

and analogously $\delta_{{\mathrm{Fe}}^{2+}}$ (for FeO) as the differences of the total energies per Fe atom in the compounds for a fixed difference between a higher ($U_{>}$) and a lower *U* value ($U_{<})$. By applying higher *U* values, the correlated electrons, which become too delocalized by LDA or GGA, are forced to a higher localization, which increases the energy of the system due to Coulomb repulsion. Therefore, $\delta_{{\mathrm{Fe}}^{3+}}>$ 0 and $\delta_{{\mathrm{Fe}}^{2+}}>$ 0.

With higher oxidation states, the number of correlated electrons is increased, so *U* has a stronger influence on the change in the energy: $\delta_{{\mathrm{Fe}}^{3+}}>\delta_{{\mathrm{Fe}}^{2+}}$. A comparison of the reaction energies for different *U* values then leads to:

(A4) $$E_{U_{>}}^{\left(r\right)}-E_{U_{<}}^{\left(r\right)}=2\left(\delta_{{\mathrm{Fe}}^{3+}}-\delta_{{\mathrm{Fe}}^{2+}}\right)>0.$$

This implies, that oxidation reaction energies increase with the increasing *U*, which is confirmed by our results for iron oxides and by the results of Wang et al. [13] for vanadium, chromium, manganese, iron, cobalt, nickel, and copper oxides.

## Appendix C: Comparison of Methods for the Determination of *U*

In this section, two conceptually different approaches are explicitly described to derive an optimal *U* value by comparing the experimental and calculated formation and oxidation energies of iron oxides. The first method is equivalent to the procedure worked out in Section 3.2, except that now only the energies of the binary iron oxide phases are taken into account. The function to be minimized with respect to *U* is:

(A5) $$f\left(U\right)=\overset{3}{\underset{i=1}{\sum }}{\left[\Delta e_{i}\left(U\right)-\Delta e_{i}^{\mathrm{fit},0}\left(U\right)\right]}^{2}.$$

with Δe as defined in Equation (2). For the fitting, the linear functions $\Delta e^{\mathrm{fit},0}$ through the origin are chosen, since the difference between $\Delta e^{\mathrm{fit},0}$ and the general linear fit $\Delta e^{\mathrm{fit}}$ is insignificant in the *U* region where f is minimal (see Figure 2a). The sum runs over Fe_2_O_3_, Fe_3_O_4_, and FeO. With $x_{\mathrm{Fe}}\equiv x,$ the fit lines can be expressed as:

(A6) $$\Delta e_{i}^{\mathrm{fit},0}\left(U\right)=x_{i}m_{0}\left(U\right)=x_{i}\frac{\sum_{j}x_{j}\Delta e_{j}\left(U\right)}{\sum_{j}x_{j}^{2}}.$$

By expressing the energy differences as linear functions of *U*: $\Delta e_{i}\left(U\right)=a_{i}U+b_{i}$, only the parameters $x_{i}$, $a_{i}$, and $b_{i}$ enter the function f(U). Minimization leads to:

(A7) $$U_{\min}=-\frac{\sum_{i}\left(a_{i}-\alpha x_{i}\right)\left(b_{i}-\beta x_{i}\right)}{\sum_{i}{\left(a_{i}-\alpha x_{i}\right)}^{2}},$$

with $\alpha :=\underset{i}{\sum }x_{i}a_{i}/x_{i}^{2}$ and $\beta :=\underset{i}{\sum }x_{i}b_{i}/x_{i}^{2}$.

In the approach by Wang et al. [13], the energies of the oxidation reactions

$$(r1)\;2\;\mathrm{FeO}+0.5{\;O}_{2}\to {\mathrm{Fe}}_{2}O_{3}\;(r2)\;3\;\mathrm{FeO}+0.5{\;O}_{2}\to {\mathrm{Fe}}_{3}O_{4}$$

are compared to the experimental values. These reaction equations can be obtained by linear combinations of the formation equations of the compounds from the pure elemental phases. For example, subtracting the reaction $\mathrm{Fe}+0.5{\;O}_{2}\to \mathrm{FeO}$ twice from $2\;\mathrm{Fe}+1.5{\;O}_{2}\to {\mathrm{Fe}}_{2}O_{3}$ yields (r1). The corresponding energies can be combined accordingly. The oxidation energies per atom ($e^{\left(r1\right)}\;\mathrm{and}\;e^{\left(r2\right)}$) are related to the formation energies of the oxides from the elemental phases per atom ($e_{i}$) via:

(A8) $$\left(\begin{array}{c} e^{\left(r1\right)}\\ e^{\left(r2\right)} \end{array}\right)=\left(\begin{array}{ccc} 1 & 0 & -4/5\\ 0 & 1 & -6/7 \end{array}\right)\left(\begin{array}{c} e_{1}\\ e_{2}\\ e_{3} \end{array}\right):=T\left(\begin{array}{c} e_{1}\\ e_{2}\\ e_{3} \end{array}\right),$$

if i=1,2,3 stands for Fe_2_O_3_, Fe_3_O_4_, and FeO, respectively. Since this relation holds for the calculated, as well as for the experimental, values, it can be formulated for their respective differences $\Delta e^{\left(r1,r2\right)}$ and $\Delta e_{1,2,3}$. Note that changing the energies $e_{1,2,3}$ by any correction proportional to the number of Fe atoms involved cancels out and has no effect on $e^{\left(r1,r2\right)}$, so it is irrelevant whether or not the correction of the combined DFT and DFT+*U* data described in Section 3.2 is considered here. Therefore, we can apply the linear dependencies $\Delta e_{i}\left(U\right)=a_{i}U+b_{i}$ with the same set of parameters as above in the first approach, which leads to Equation (A7).

The function to be minimized to find the closest match between the calculated and experimental oxidation energies is:

(A9) $$g\left(U\right)={\left[\Delta e^{\left(r1\right)}\left(U\right)\right]}^{2}+{\left[\Delta e^{\left(r2\right)}\left(U\right)\right]}^{2}.$$

With the definitions $\vec{a}≔\left(a_{1},a_{2},a_{3}\right)$ and $\vec{b}≔\left(b_{1},b_{2},b_{3}\right)$, the *U* value where g is minimal can be found as:

(A10) $$U_{\min}=-\frac{\left(T\vec{a}\right)\cdot \left(T\vec{b}\right)}{{\left(T\vec{a}\right)}^{2}},$$

with the matrix T as defined in Equation (A8).

From the data points $\Delta e_{i}\left(U\right)$ shown in Figure 2a, one obtains $\vec{a}=\left(0.132,\;0.153,\;0.105\right)$ eV/*U* and $\vec{b}=\left(0.198,\;0.209,\;0.523\right)$ eV, leading to $U_{\min}$= 4.00 eV and $U_{\min}$= 4.05 eV for the first (Equation (A7)) and second approach (Equation (A10)), respectively. The functions f(U) and g(U) are shown in Figure A1, indicating that the two approaches are indeed different from each other, even though they use the same set of calculated and experimental input data and lead to very close minima.

## Appendix D: Phase Diagram with Respect to the Precursor Compounds

This section explicitly describes for the example of LaFeO_3_ (LFO), how a phase diagram can be derived with respect to reservoir energies of binary compounds and molecular oxygen instead of the usual representation considering only the elemental phases. The latter corresponds to a hypothetical solid-state reaction La + Fe + (3/2)O_2_ → LaFeO_3_. In a more realistic synthesis scenario, stable oxide compounds for LFO, e.g., La_2_O_3_ and Fe_2_O_3_, are mixed by mechanical and/or chemical treatment and exposed to an oxygen atmosphere [53,54]. In an analogy to Equations (1) and (3), $E_{c}^{\mathrm{form}}$ given in Equation (A11) expresses the formation energy of LFO with respect to the binary oxides in their ground-state phases, which are again labeled by the superscript (0):

(A11) $$E_{c}^{\mathrm{form}}\left(\mathrm{LFO}\right)=E^{\mathrm{total}}\left(\mathrm{LFO}\right)-\frac{1}{2}\left[\mu^{\left(0\right)}\left({\mathrm{La}}_{2}O_{3}\right)+\mu^{\left(0\right)}\left({\mathrm{Fe}}_{2}O_{3}\right)\right]=\frac{1}{2}\Delta \mu \left({\mathrm{La}}_{2}O_{3}\right)+\frac{1}{2}\Delta \mu \left({\mathrm{Fe}}_{2}O_{3}\right).$$

Since $E_{c}^{\mathrm{form}}\left(\mathrm{LFO}\right)$ has a fixed value, the second equation of Equation (A11) contains only one free variable, which implies that fixing the value of one chemical potential, e.g., $\Delta \mu \left({\mathrm{La}}_{2}O_{3}\right)$ (i.e., setting the conditions in this compound in terms of rich or poor), defines the chemical potential/conditions of the other compound ($\Delta \mu \left({\mathrm{Fe}}_{2}O_{3}\right))$. However, processing under an oxygen atmosphere results in a constant exchange of oxygen atoms between the compound phases and the atmospheric reservoir, which also produces free non-oxygen elements (La and Fe) at certain energies. This can be formally taken into account by expressing one of the chemical potentials of the precursor compounds, say $\mu \left({\mathrm{Fe}}_{2}O_{3}\right)$, by the respective energies of its elemental components, leading to:

(A12) $$\Delta \mu \left({\mathrm{Fe}}_{2}O_{3}\right)=\mu \left({\mathrm{Fe}}_{2}O_{3}\right)-\mu^{\left(0\right)}\left({\mathrm{Fe}}_{2}O_{3}\right)=2\mu \left(\mathrm{Fe}\right)+3\mu \left(O\right)-\mu^{\left(0\right)}({\mathrm{Fe}}_{2}O_{3})\;=\;2\left[\mu^{\left(0\right)}\left(\mathrm{Fe}\right)+\Delta \mu \left(\mathrm{Fe}\right)\right]+3\left[\mu^{\left(0\right)}\left(O\right)+\Delta \mu \left(O\right)\right]-\mu^{\left(0\right)}\left({\mathrm{Fe}}_{2}O_{3}\right).$$

With this expression, Equation (A11) can be reformulated:

(A13) $$\Delta \mu \left({\mathrm{La}}_{2}O_{3}\right)+2\Delta \mu \left(\mathrm{Fe}\right)+3\Delta \mu \left(O\right)=2E_{c}^{\mathrm{form}}\left(\mathrm{LFO}\right)+E^{\mathrm{form}}\left({\mathrm{Fe}}_{2}O_{3}\right),$$

with $E^{\mathrm{form}}\left({\mathrm{Fe}}_{2}O_{3}\right)$ according to Equation (1) (note that $\mu^{\left(0\right)}\left({\mathrm{Fe}}_{2}O_{3}\right)=E^{\mathrm{total}}\left({\mathrm{Fe}}_{2}O_{3}\right)$ for the ground-state phase).

Equation (A13) now defines the phase space of LFO with the free variables $\Delta \mu \left({\mathrm{La}}_{2}O_{3}\right)$ and Δμ(O), which can be visualized in a two-dimensional diagram, e.g., with axes $x=\Delta \mu \left({\mathrm{La}}_{2}O_{3}\right)$ and y=Δμ(O). Following Equation (A11), an appropriate second x-axis can then be chosen as $x^{\prime }=\Delta \mu \left({\mathrm{Fe}}_{2}O_{3}\right)=2E_{c}^{\mathrm{form}}\left(\mathrm{LFO}\right)-x$. Lines y(x) constraining the phase space of LFO with respect to other phases ${\mathrm{La}}_{l}{\mathrm{Fe}}_{m}O_{n}$ can be obtained by expressing Δμ(La) and Δμ(Fe) in the formation energy of ${\mathrm{La}}_{l}{\mathrm{Fe}}_{m}O_{n}$ according to Equation (3) by x and y. For example, Equation (A13) yields $\Delta \mu \left(\mathrm{Fe}\right)=E_{c}^{\mathrm{form}}\left(\mathrm{LFO}\right)+0.5\;E^{\mathrm{form}}\left({\mathrm{Fe}}_{2}O_{3}\right)-0.5x-1.5y$. On the other hand, expressing $\Delta \mu \left({\mathrm{La}}_{2}O_{3}\right)$ by the elemental chemical potentials in analogy to Equation (A12) and using Equation (A11) leads to $\Delta \mu \left(\mathrm{La}\right)=0.5\;E^{\mathrm{form}}\left({\mathrm{La}}_{2}O_{3}\right)+0.5x-1.5y$. It then follows from Equation (3):

(A14) $$x\left[-l+m\right]+y\left[3l+3m-2n\right]=K_{lmn},$$

with

(A15) $$K_{lmn}:=2mE_{c}^{\mathrm{form}}\left(\mathrm{LFO}\right)+lE^{\mathrm{form}}\left({\mathrm{La}}_{2}O_{3}\right)+mE^{\mathrm{form}}\left({\mathrm{Fe}}_{2}O_{3}\right)-2E^{\mathrm{form}}\left({\mathrm{La}}_{l}{\mathrm{Fe}}_{m}O_{n}\right).$$

For example, the line in the phase diagram separating LFO from elemental Fe (l=n=0, m=1, $E^{\mathrm{form}}\left(\mathrm{Fe}\right)=0$) is given by $x+3y=E_{c}^{\mathrm{form}}\left(\mathrm{LFO}\right)+E^{\mathrm{form}}\left({\mathrm{Fe}}_{2}O_{3}\right)$, and the line between LFO and $O_{2}$ by y=0 is consistent with the definition of y. The formation energies used in Equations (A13) and (A15) are calculated with the corrections described in Section 2.3 (Equation (4)) and Section 2.4 (Equation (6)).

The procedure described here for LFO is transferable to other solid-state processing routes and arbitrary ternary compounds, such as Li_5_FeO_4_ and NaFeO_2_ considered in this study. Note that the chemical potentials of the elemental phases need to be included in the formulation of the formation energy with respect to the compound phases ($E_{c}^{\mathrm{form}}$) corresponding to Equation (A11) if the elemental phases are part of the reaction due to the stoichiometric constraints (see Section 3.5.2 and Section 3.5.3). This, however, does not change the methodology following Equations (A12)–(A15). For example, the starting point for deriving the phase diagram of Li_5_FeO_4_ with respect to $\Delta \mu \left({\mathrm{Li}}_{2}O\right)$ and $\Delta \mu \left(O_{2}\right)$ would be in analogy to Equation (A11):

(A16) $$E_{c}^{\mathrm{form}}\left({\mathrm{Li}}_{5}{\mathrm{FeO}}_{4}\right)=4\Delta \mu \left({\mathrm{Li}}_{2}O\right)+\Delta \mu \left(\mathrm{Fe}\right)-3\Delta \mu \left(\mathrm{Li}\right),$$

according to the reaction $4{\;\mathrm{Li}}_{2}O+\mathrm{Fe}⟶{\mathrm{Li}}_{5}{\mathrm{FeO}}_{4}+3\;\mathrm{Li}$. The procedure then follows the steps described above. However, in contrast to the situation for LaFeO_3_ and Equation (A11), Equation (A16) contains two free variables. Therefore, choosing $\Delta \mu \left({\mathrm{Li}}_{2}O\right)$ as the horizontal (x)-axis of the phase diagram does not allow for an alternative horizontal axis $x^{\prime }=\Delta \mu \left(\mathrm{Fe}\right)$ or $x^{\prime }=\Delta \mu \left(\mathrm{Li}\right)$. Instead, these quantities can be represented by height functions in the phase diagram, and, e.g., illustrated by color coding, as shown in Figure 5 and Figure 6.
